# Responses of soil bacterial community structure to different artificially restored forests in open-pit coal mine dumps on the loess plateau, China

**DOI:** 10.3389/fmicb.2023.1198313

**Published:** 2023-07-28

**Authors:** Shuang Liu, Yuru Gao, Jianwen Chen, Junjian Li, Hong Zhang

**Affiliations:** ^1^Institute of Loess Plateau, Shanxi University, Taiyuan, China; ^2^College of Environment and Resources, Shanxi University, Taiyuan, China

**Keywords:** vegetation restoration, forest types and ages, soil properties, microbial community, mining area

## Abstract

Artificial vegetation restoration is an effective method for improving soil quality. In areas experiencing coal mine subsidence, the microbial community is essential for reconstructing the ecological balance of the soil. Studies are needed to examine how soil microbial community structure respond to different artificial forest restoration types and ages, especially over long-term periods. Therefore, in this study, 10, 20, and 30-year trials were chosen with two restoration types: *Pinus tabuliformis* (PT) and *Ulmus pumila* (UP). The objective was to determine how various types and ages of forest restoration affect the structure of soil bacterial communities, as well as the soil environmental factors driving these changes. The results showed that artificial 30-year restoration for both PT and UP can improve soil physical and chemical properties more than restoration after 10 and 20 years. The soil bacterial community structure remarkably differed among the different forest types and restoration ages. The bacterial diversity was higher in UP than in PT; the alpha diversity at longer restoration years (30 and 20) was significantly higher than at 10 years for both PT and UP. Moreover, soil nutrients and pH were the primary soil environmental factors driving bacterial community structure in the PT and UP. Finally, the integrated fertility index (IFI) at 30 years of restoration was considerably higher for PT and UP, and thus, is more beneficial to the restoration of soil after coal mining. Our findings are useful for studying improvement in soil quality and the restoration of the ecological environment in mining areas.

## Introduction

The loess plateau in China is one of the unique landforms inf the world. In this region, the soil and plant ecosystems are extremely fragile and sensitive, especially after surface coal mining and land reclamation (Bai et al., 2006; Liu et al., 2016; Zhao et al., 2023). With coal open-pit mining, the vegetation is removed from the land surface, which changes the landscape and landforms, alters the soil’s characteristics and structure, and drastically disrupts soil hydrological regimes through soil excavation (Shrestha and Lal, 2011; Zhao et al., 2013; Wang et al., 2014; Ahirwal and Maiti, 2016). Mining also decreases the organic carbon and associated nutrients in the soil (Zhang et al., 2015), making coal mining the most prominent example of ecosystem degradation caused by humans (Zhang et al., 2015; Liu et al., 2016). In China, Shanxi province has abundant mineral resources, but the long-term excessive exploitation of these resources has seriously damaged the ecosystem in coal mining areas, especially in the north Shanxi coal area, which is located in a semiarid ecological zone in the loess hilly area.

The dumps containing mining waste are environmentally devastated areas: these areas constitute the land most damaged by open-pit mining (Chen et al., 2020). Currently, mine dumps are required to be restored following the completion of mining activities, because open-pit coal mining is only a temporary land use (Mukhopadhyay et al., 2017). Therefore, a number of vegetation restoration activities are implemented to restore the environment around coal mine waste dumps, including tree planting, agricultural reclamation, and constructing botanical gardens (Huang and Liu, 2013). Selecting the appropriate vegetation restoration is crucial for maintaining and enhancing the stability of the artificial restoration soil ecosystem in mining areas (Tripathi et al., 2016; Li and Liber, 2018) because the vegetation on mine dumps helps to prevent desertification, reduce water and nutrient runoff, and reduce soil erosion (Mukhopadhyay et al., 2017; Chen et al., 2020). However, the disturbed mining land usually has a compacted structure, a disorganized stratigraphic sequence, complex surface material, and impaired soil qualities, which hinder the ability of natural vegetation to restore the ecosystem (Zhang et al., 2015; Zhou et al., 2017). Additionally, the process of restoring ecosystem functions through natural vegetation regeneration is slow compared with using artificially restored vegetation that planted and managed by human (Li and Liber, 2018). With proper management of vegetation restoration, however, restoring disturbed mined soils is possible (Shrestha and Lal, 2010).

Artificial restoration of vegetation, implemented through human intervention, is the most effective and useful implementation of biological methods to establish appropriate vegetation in mining areas (Josa et al., 2012; Xu, 2012). Different vegetation types and restoration periods can lead to different organic matter and carbon contents, which cause changes in the physical structure, chemical composition, and microbiological characteristics of the soil (Fu et al., 2010; Nawaz et al., 2013; Zhao et al., 2013). For example, the soil bulk density decreases when the soil porosity increases due to root penetration (Yang and Wang, 2013); thus soil infiltration and field capacities (Cejpek et al., 2013) increase. In addition, the soil pH decreases (Zipper et al., 2013) and the soil total nitrogen (TN) and soil organic carbon (SOC) contents increase (Shrestha and Lal, 2010) as the restoration age increases. The microbial community is sensitive to soil conditions and connected to a variety of soil processes, such as nitrogen cycling and the breakdown of organic waste (Kaschuk et al., 2010; Liang et al., 2021). Researchers are increasingly using microbial properties as an ecological indicator of the effects of soil recovery efforts in mining areas (Helingerova et al., 2010; Claassens et al., 2012) because the activity of soil organisms provides useful information when monitoring the quality of the soil produced from intensive coal mining activities (Li et al., 2015; Zhao et al., 2023).

Presently, ecological restoration, vegetation reconstruction, and soil reclamation are still in the initial stages in most coal mining areas in Shanxi (Li and Liber, 2018). As such, how the features of soil microbial communities in open-pit coal mine dumps under loess environments respond to different ages and types of artificial forest restoration remains unknown. In addition, the effect of ecosystem restoration practices after disturbances also requires observation over long time periods. It is especially important to obtain information on the effects of long-term vegetation restoration activities on the establishment of forests and the associated soil development in reclaimed coal mine dumps in Shanxi, China. Therefore, a study about the changes in the soil microbial community observed with different types and ages of artificial forest restoration in waste dumps is especially important for directing future ecological restoration.

The largest open-pit coal mine in China is the Antaibao mine, situated in the Pingshuo mining area of Shanxi, and is a representative mine in the area. The Antaibao mine has three waste dumps (southern, western, and inner open-pit coal mine dumps). Focusing on these mine dumps, this study examined the relationships between soil environmental factors and soil bacterial community characteristics under different forest types and ages. The hypotheses were proposed that the soil microbial community structure of both coniferous and broad-leaf forests would differ after different restoration periods, and the bacterial community structure of broad-leaf forests would differ from those of coniferous forests. The specific objectives of this study were (1) to assess the impact of different forest restoration types and ages on soil physicochemical properties and bacterial communities, and assess their impact factors, and (2) to explore the driving effect of the relationship between soil physicochemical properties and bacterial communities under long-term restoration, and provide a scientific basis for the management of vegetation restoration activities in open-pit coal mine dumps in loess areas in China.

## Materials and methods

### Study area and sampling processes

**Figure 1:**
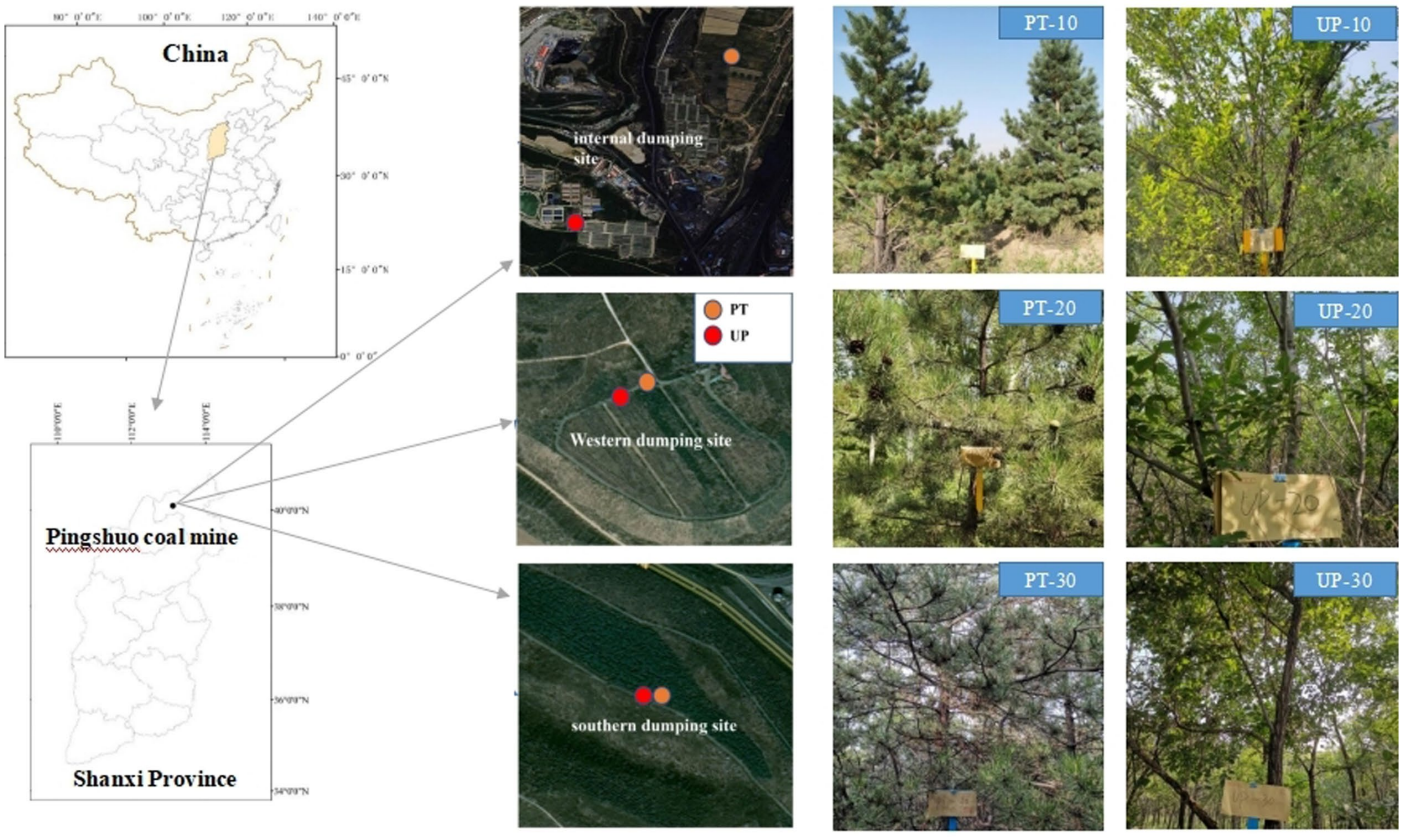
Location of study area and soil sampling sites in the Pingshuo coal mining area of Shanxi province. PT, *Pinus tabuliformis*; UP, *Ulmus pumila*.

The Pingshuo mining region (112°10′–112°30′E, 37°26′–37°36′N) is situated in Shuozhou city, north of Shanxi province in China (Figure 1). The area has a temperate, semiarid continental monsoon climate. The mean annual precipitation is 428.2–449.0 mm, and the mean annual temperature ranges from 4.8°C to 7.8°C. The geomorphic type of the mining area is classified as loess low mountains and hills. Chestnut soil, which is characterized by a low organic matter content and poor structure and is categorized as an chestnut soil that predominates in this area (Zhou et al., 2017). The total area of the Pingshuo mine is approximately 160 km^2^; the west, south, and inner dumps of the Antaibao open-pit coal mine restoration area were chosen as the study area. The dump location information is provided in Figure 1.

All the soil samples were collected in the summer of 2020; Figure 1 shows the soil sampling locations at the three dumps for both PT and UP restoration. Soil samples were collected from a soil depth of 0–10 cm, and all the samples were obtained at five random sampling sites with five replicates for each forest type and restoration year. We created one composite sample that comprised all 25 subsamples, which we then placed into polyethylene (PE) bags. One kilogram of soil was divided into two parts: the fresh half was kept in an icebox and transported to the laboratory within 24 h and then kept at −80°C for DNA extraction; the other part was air-dried, crushed, passed through a 2.00 mm sieve for the determination of soil chemical properties.

### Measurement of soil properties

Soil water content (SWC) was calculated using the oven-drying method (Evett, 2008). The cutting ring method was used to analyze the soil bulk density (BD) (Zeng et al., 2014; Liu et al., 2022a). A pH meter was used to determine the pH of the soil at a soil-to-water ratio of 1:2.5 (Zhao et al., 2023). Using a vario MACRO cube and a multi N/C 3100 (Analytik Jena GmbH, Jena, Germany), the total carbon (TC) and total nitrogen (TN) contents were calculated. Following digestion, the percentage of soil total phosphorus (TP) was determined using NaOH melting molybdenum antimony colorimetric analysis. After extraction with 0.5 M NaHCO_3_ at pH 8.5, the available P (AP) was measured using the molybdate–ascorbic acid technique (Zhen et al., 2015). After extraction with 2.0 mol L^−1^ KCl, the ammonia nitrogen (NH_4_^+^-N) and nitrate nitrogen (NO_3_^−^-N) contents were quantified using a flow injection auto analyzer (Dechem-Tech.G.mblt, Germany; Wu et al., 2021).

### Soil DNA extraction and quantitative PCR analysis

According to the manufacturer’s instructions, 0.5 g of soil material was weighed to extract total DNA from the soil using a TIANnamp Soil DNA Kit (TIANGEN, China). The highly variable V3–V4 region of the bacterial 16S rRNA gene was amplified using primers 338 F (5′-ACTCCTACGGGAGGCAGCAG-3′) and 806 R (5′-GGACTACHVGGGTWTCTAAT-3′; Dennis et al., 2013). Each sample was subjected to the following amplification process: initial denaturation at 95°C for 3 min, followed by 27 rounds of denaturation at 95°C for 30 s, annealing at 55°C for 30 s, extension at 72°C for 45 s, and a final extension at 72°C for 10 min (Yu et al., 2018). The PCR products were purified, and an Illumina MiSeq PE300 was used to conduct high-throughput paired-end sequencing (Allwegene Co., Ltd., Beijing, China).

### Processing of sequencing data

The raw data from the Illumina MiSeq sequencing was examined using the QIIME software suite (version 1.9.1). Using Usearch (version 11, available at http://drive5.com/uparse/), the operational taxonomic units (OTUs) were identified at a 97% similarity level (Wang et al., 2022), and an OTU table was generated for each sample for statistical analysis. The Shannon, ACE, and Chao1 diversity indices were calculated using the Mothur program (version 1.30.2; available for download at https://www.mothur.org/wiki/Download_mothur; Schloss et al., 2009). Each representative sequence’s taxonomic designation was established using Silva (version 138, available at https://www.arb-silva.de/). The Ribosomal Database Project (RDP) Classifier (version 2.13, available at http://sourceforge.net/projects/rdpclassifier/) identified the taxonomic identity of the phylotypes with a 70% level of confidence.

### Statistical analysis

One-way and two-way analysis of variance (ANOVA) were applied to evaluate the effects of the forest type and restoration age on the soil’s physicochemical properties and alpha diversity of the soil’s bacterial community. The least-significant differences (LSD) test was used for analysis of variance at the 0.05 level (IBM SPSS Statistics 26.0). Gephi 0.92 was applied to display co-occurrence network that using the OTUs after screening with the statistical indicators Pearson’s coefficient r > 0.80 and a *p-*value of 0.001. Spearman correlation was used to analyzed the relationship between soil’s physicochemical properties and bacterial community. The correlation heat map was analyzed using Wekemo Bioincloud.^1^ Redundancy analysis (RDA) and Monte Carlo permutation tests was used to analyze soil environmental factors effect on the bacterial community. Variation-partitioning analysis (VPA) and partial Mantel test were used to separately determine the relative contribution of restoration age and soil environmental factors to microbial community variation. RDA, Monte Carlo permutation tests, VPA and Partial Mantel test were all conducted using the “vegan” package in R 4.2.1 (Dixon, 2003). The co-occurrence network analyses were visualized with Gephi 0.9.7.^2^ IBM SPSS Amos 26.0 was used to establish the structural equation model (SEM). The chi-squared test (*p* > 0.05), root mean square error of approximation (RMSEA) < 0.05, comparative fit index (CFI) > 0.95, and goodness-of-fit index (GFI) > 0.95 were used to assess model fitness (Chen et al., 2019).

To determine the mine soil integrated fertility index (IFI), we used principal component analysis (PCA) to select the soil physicochemical and microbial characteristics; the soil quality was described by the principal components (PCs) with an eigenvalue ≥1 which explained at least 5% of the variation in the data (Kaiser, 1960; Wander and Bollero, 1999; Mukhopadhyay et al., 2014). The integrated fertility index (IFI) can be calculated as follows:


$$IFI=\overset{n}{\underset{i=1}{\sum }}WiSi$$

where *W* is the ith PC eigenvalue; and *S* is the contribution rate of the ith indicator for each variable, *i* = 1, 2, 3…*n*.

## Results

### Soil physical and chemical properties

For soil physical properties, the soil water content (SWC) of PT-30 soil was significantly higher than that of PT-20 and PT-10 soils, and the soil bulk density (BD) of PT-30 soil was significantly lower than that of PT-20 and PT-10 soils (*p* < 0.05). We found no significant difference (*p* > 0.05) between PT-20 and PT-10 soils. Similar results were observed for UP, e.g., UP-30 had a significantly (*p* < 0.05) higher SWC and lower soil BD than UP-20 and UP-10 soils; we also observed no significant difference (*p* > 0.05) between UP-20 and UP-10 soils (Table 1). In terms of soil chemical properties, the soil pHs of PT-30 and UP-30 were both significantly (*p* < 0.05) lower than those at 20 and 10 years. At 30 years, PT and UP both had the highest soil TC, TN, TP, NO_3_^−^-N, and NH_4_^+^-N contents compared with those at 20 and 10 years. The AP content also increased as restoration age increased for UP, but decreased as restoration age increased for PT (Table 1).

**Table 1:** Soil physicochemical properties for different restoration ages.

Forest type	Restoration age	Soil water content	Bulk density	pH	TC	T N	TP	NO_3_^−^-N	NH_4_^+^-N	AP
		(g·g^−1^)	(g·cm^−3^)		(g·kg^−1^)	(g·kg^−1^)	(g·kg^−1^)	(mg∙kg^−1^)	(mg∙kg^−1^)	(mg∙kg^−1^)
PT	10 years	0.09 ± 0.01b	0.48 ± 0.01a	7.95 ± 0.01a	11.72 ± 0.68c	0.27 ± 0.02c	0.69 ± 0.04ab	10.39 ± 0.6c	8.10 ± 0.47b	8.90 ± 0.51a
	20 years	0.06 ± 0.01b	0.51 ± 0.02a	7.95 ± 0.01a	20.98 ± 1.21b	1.02 ± 0.06b	0.61 ± 0.04b	13.45 ± 0.78b	9.53 ± 0.55ab	7.90 ± 0.46ab
	30 years	0.24 ± 0.05a	0.44 ± 0.01b	7.84 ± 0.01b	35.84 ± 2.07a	1.65 ± 0.1a	0.78 ± 0.05a	21.25 ± 1.23a	10.69 ± 0.62a	6.80 ± 0.39b
UP	10 years	0.1 ± 0.03b	0.49 ± 0.01a	8.00 ± 0.00a	31.23 ± 1.8b	1.75 ± 0.1b	0.65 ± 0.04b	11.22 ± 0.65a	10.15 ± 0.59b	4.60 ± 0.27b
	20 years	0.11 ± 0.02b	0.48 ± 0.02a	7.81 ± 0.01b	32.65 ± 1.89ab	1.9 ± 0.11b	0.75 ± 0.04ab	11.75 ± 0.68a	11.83 ± 0.68ab	7.70 ± 0.44a
	30 years	0.27 ± 0.01a	0.39 ± 0.01b	7.75 ± 0.02c	35.10 ± 2.03a	2.31 ± 0.13a	0.87 ± 0.05a	11.78 ± 0.68a	13.01 ± 0.75a	8.80 ± 0.51a
**Summary of ANOVA (*p*-values)**										
Forest type		0.02	0.008	<0.001	<0.001	<0.001	0.004	<0.001	<0.001	0.001
Restoration age		<0.001	<0.001	<0.001	<0.001	<0.001	<0.001	<0.001	<0.001	0.001
Forest type × restoration age		0.255	0.009	<0.001	<0.001	<0.001	0.002	<0.001	0.923	<0.001

PT, *Pinus tabuliformis*; UP, Ulmus pumila; TC, total carbon content; TN, total nitrogen content; TP, total phosphorus content; NO_3_^−^-N, nitrate nitrogen; NH_4_^+^-N, ammonium nitrogen; AP, available P content. Values are mean ± standard deviation (*n* = 5). Statistical significance was assessed by one-way ANOVA followed by LSD multiple comparison test. Different lowercase letters per column indicate the significant difference (*p* < 0.05).

### Bacterial community composition and diversity

**Figure 2:**
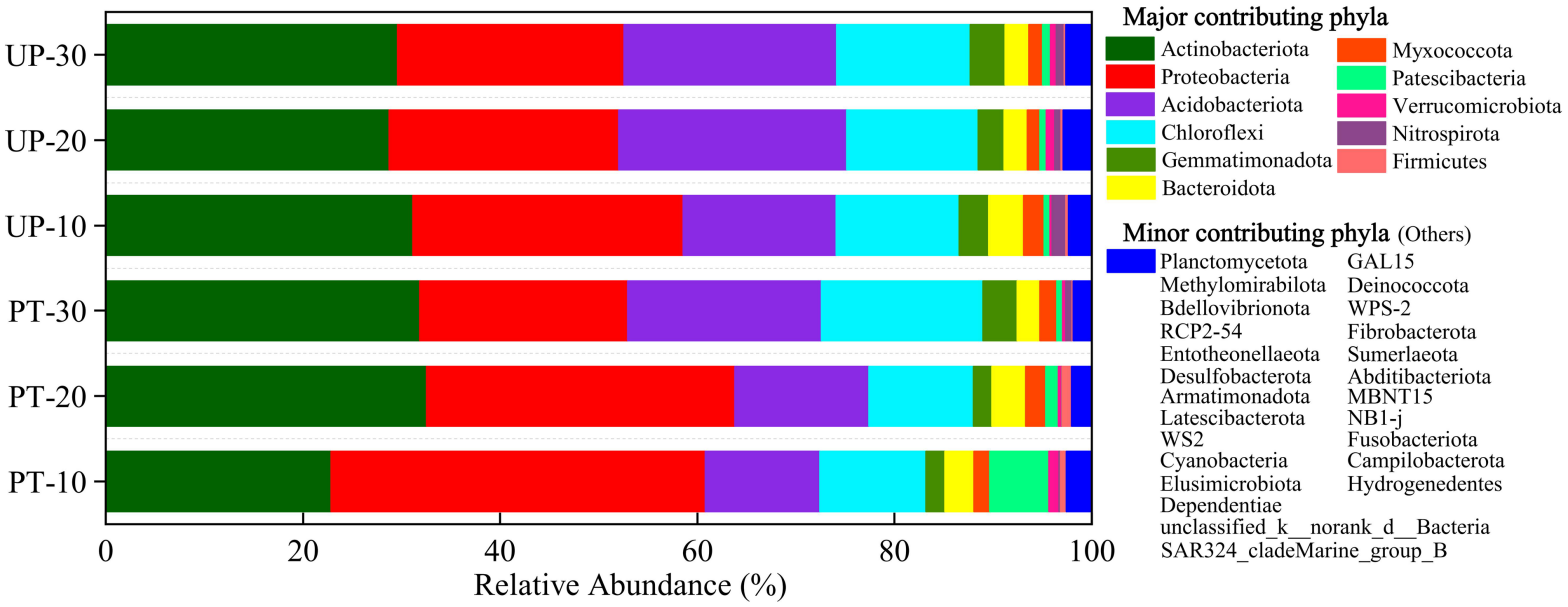
Dominant soil bacterial phyla for PT and UP after 10, 20, and 30 years of restoration. Bacterial phyla with a relative frequency of less than 0.1% are included as “others.”

The bacterial community compositions of the two forest types (PT and UP) at three restoration ages (10, 20, and 30 years) were elucidated by high-throughput sequencing analysis. The dominant bacterial phylum community groups were *Proteobacteria* (20.52%–42.09%), *Actinobacteria* (21.63%–41.33%), *Acidobacteria* (4.72%–23.73%), and *Chloroflexi* (2.90%–16.27%), whereas *Gemmatimonadetes* (0.54%–5.72%), *Bacteroidetes* (2.10–3.65%), *Myxococcota* (0.81%–2.32%), *Patescibacteria* (0.48%–1.36%), *Verrucomicrobia* (0.20%–1.78%), *Firmicutes* (0.06%–1.64%), and *Nitrospirota* (0.03%–1.38%) accounted for a small portion of the bacterial composition; the rest were rare community groups (Figure 2). The relative abundances of *Proteobacteria* in PT and UP both decreased with increasing restoration age, but for *Acidobacteria* and *Chloroflex*, a different pattern was observed in which the abundances increased with increasing restoration age. The abundance of *Actinobacteria* also increased with increasing restoration ages for PT, but we found no significant variations (*p* > 0.05) for UP among the restoration ages (Figure 2; Supplementary Table S1).

The bacterial diversity at the OTU (operational taxonomic units) level was higher in UP than in PT at all three restoration ages. For PT restoration, both PT-30 and PT-20 soils had significantly higher Shannon, ACE, and Chao1 diversity indices than PT-10 soil. For UP restoration, significantly higher values of the Shannon, ACE, and Chao1 indices for UP-30 soil were also observed. However, for the Shannon index between UP-30 and UP-20 and between UP-20 and UP-10, no significant differences were found (Table 2). In addition, the dissimilarity analysis showed significant differences in the bacterial community structures among all restoration ages for PT and UP (*p* < 0.05; Supplementary Table S2).

**Table 2:** Alpha diversity of soil bacterial community for different vegetation restoration types.

Forest type	Restoration age	Shannon	Ace	Chao1
PT	10 years	5.6431 ± 0.0115b	1640.9 ± 32.1b	1624.2 ± 38.0b
	20 years	6.0587 ± 0.0103a	2233.9 ± 46a	2269.2 ± 63.3a
	30 years	6.0706 ± 0.0104a	2260.1 ± 50.2a	2272.9 ± 65.6a
UP	10 years	5.9798 ± 0.0113b	2175.9 ± 46.1b	2181.0 ± 59.3b
	20 years	6.1762 ± 0.0105a	2282.6 ± 41.4ab	2283.4 ± 53.0ab
	30 years	6.1861 ± 0.0103a	2366.6 ± 45.7a	2393.4 ± 61.6a
**Summary of ANOVA (*P*-values)**				
Forest type		<0.001	<0.001	<0.001
Restoration age		<0.001	<0.001	<0.001
Forest type x restoration age		<0.001	<0.001	<0.001

Values are mean ± SE, *n* = 5. Statistical significance was assessed by one-way ANOVA followed by LSD multiple comparison test. Different lowercase letters in the same column indicate significant difference (*p* < 0.05).

**Figure 3:**
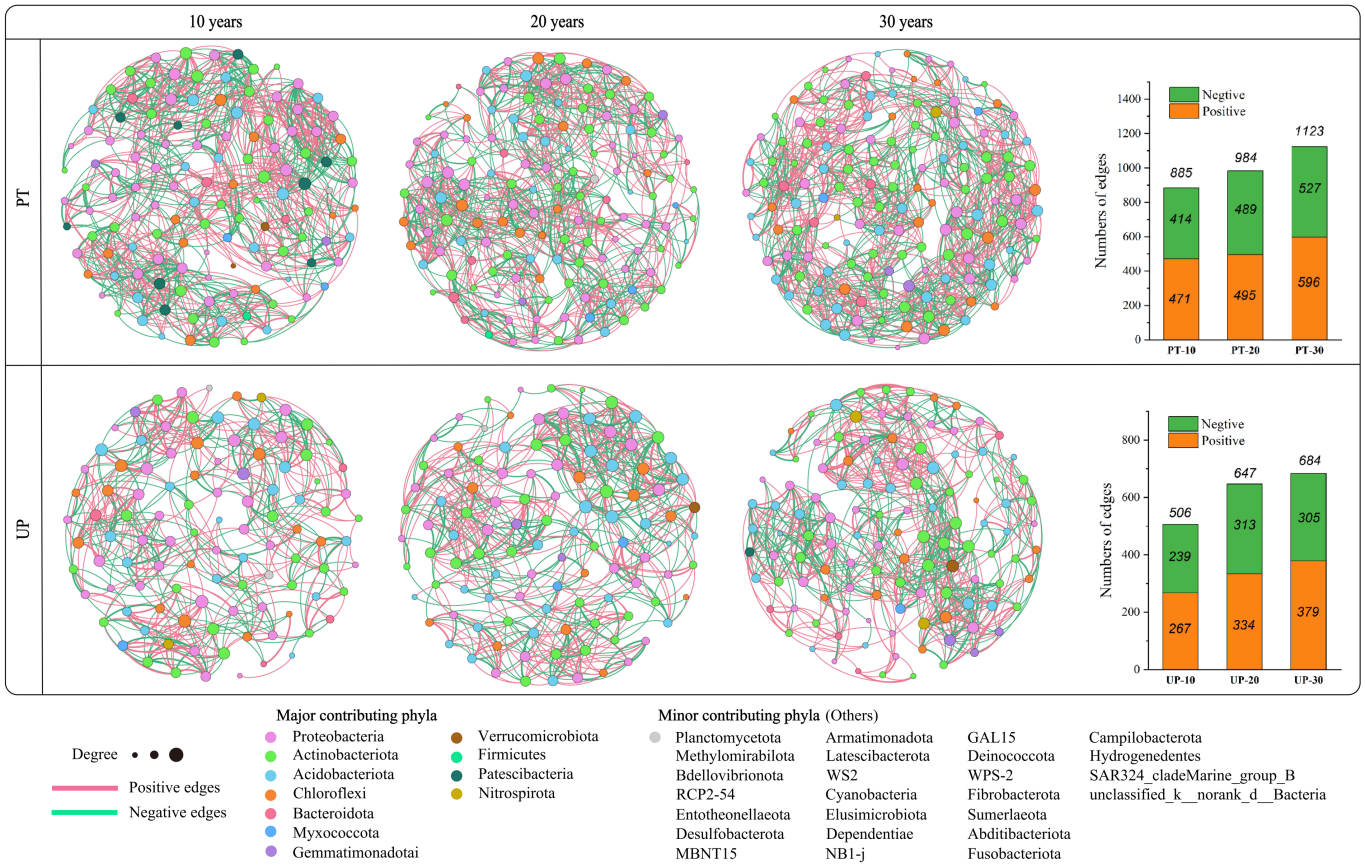
Soil bacterial community co-occurrence networks of PT and UP at restoration ages of 10, 20, and 30 years. The nodes are colored by phylum and represent an operational taxonomic unit (97% sequence identify threshold, OTU). The size of each node is proportional to the number of connections (degrees).

A co-occurrence network can be used to decipher the potential interaction patterns among bacterial species (Fan et al., 2011; Bi et al., 2021). Thus, a soil bacterial community co-occurrence network was generated based on the bacterial taxa at the OTU (operational taxonomic units) level for the restoration age of 10 to 30 years for PT and UP (Figure 3). For PT, the formed soil bacterial interaction networks in PT-10, PT-20, and PT-30 soils included 885, 984, and 1,123 edges, respectively. For UP, the formed soil bacterial interaction networks included 506, 647, and 684 edges in UP-10, UP-20, and UP-30 soils, respectively. Additionally, the proportion of positive correlations in the bacterial networks was larger than the proportion of negative correlations for both PT and UP (Figure 3).

### Relationships between bacterial community and soil properties

**Figure 4:**
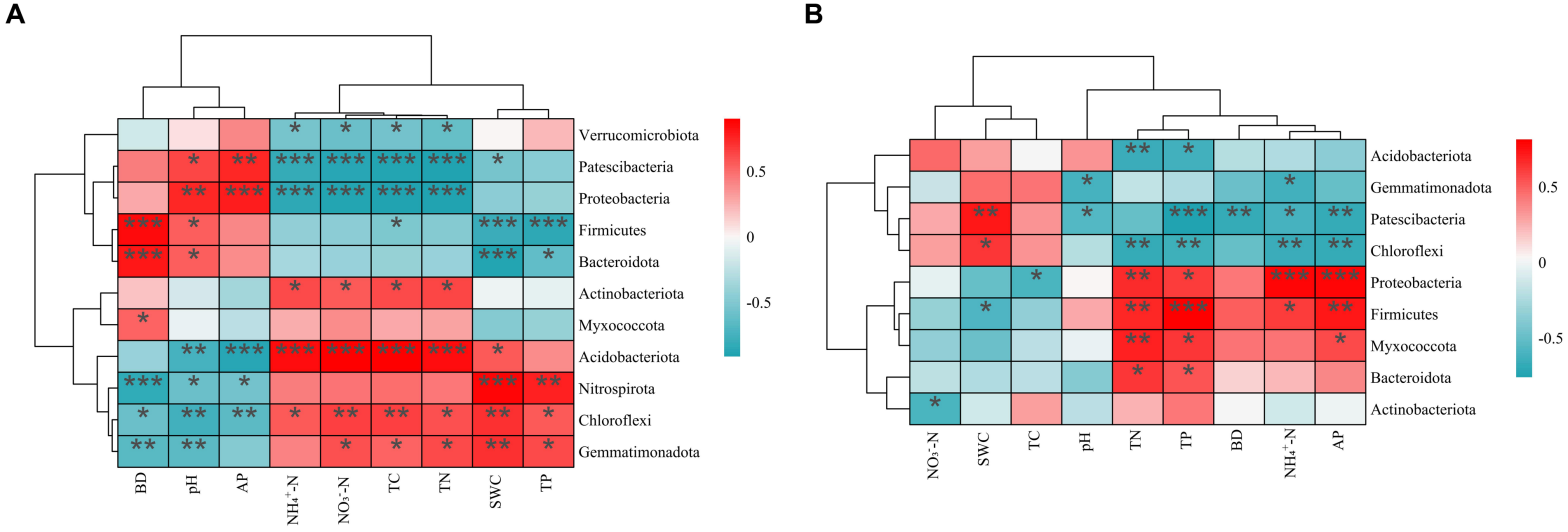
Spearman correlation heatmap: correlation between soil’s physicochemical properties and relative abundances of the dominant bacterial community (phylum level) of PT **(A)** and UP **(B)**. Significant correlations are reported as follows: ^***^p < 0.001; ^**^p < 0.01; ^*^p < 0.05.

The results of the spearman correlation test revealed that all the physical and chemical soil properties significantly correlated with some of the dominant soil bacterial phyla for both PT and UP (Figure 4). For PT, most soil properties, including SWC (soil water content), TP, NO_3_^−^-N, NH_4_^+^-N, TN, and TC contents, positively correlated with the four dominant soil bacterial phyla (*Gemmatimonadetes*, *Acidobacteria*, *Chloroflexi*, and *Nitrospirota*), but negatively correlated with three dominant phyla (*Proteobacteria*, *Firmicutes*, and *Patescibacteria*). Soil BD, pH, and TP showed the opposite correlations (Figure 4A). For UP, the contents of TP, AP, TN, and NH_4_^+^-N were significantly negatively correlated with the bacterial phyla *Proteobacteria*, *Bacteroidota*, and *Myxococcota*, but were significantly positively correlated with *Acidobacteria*. The soil BD and pH showed the opposite correlations (Figure 4B).

**Figure 5:**
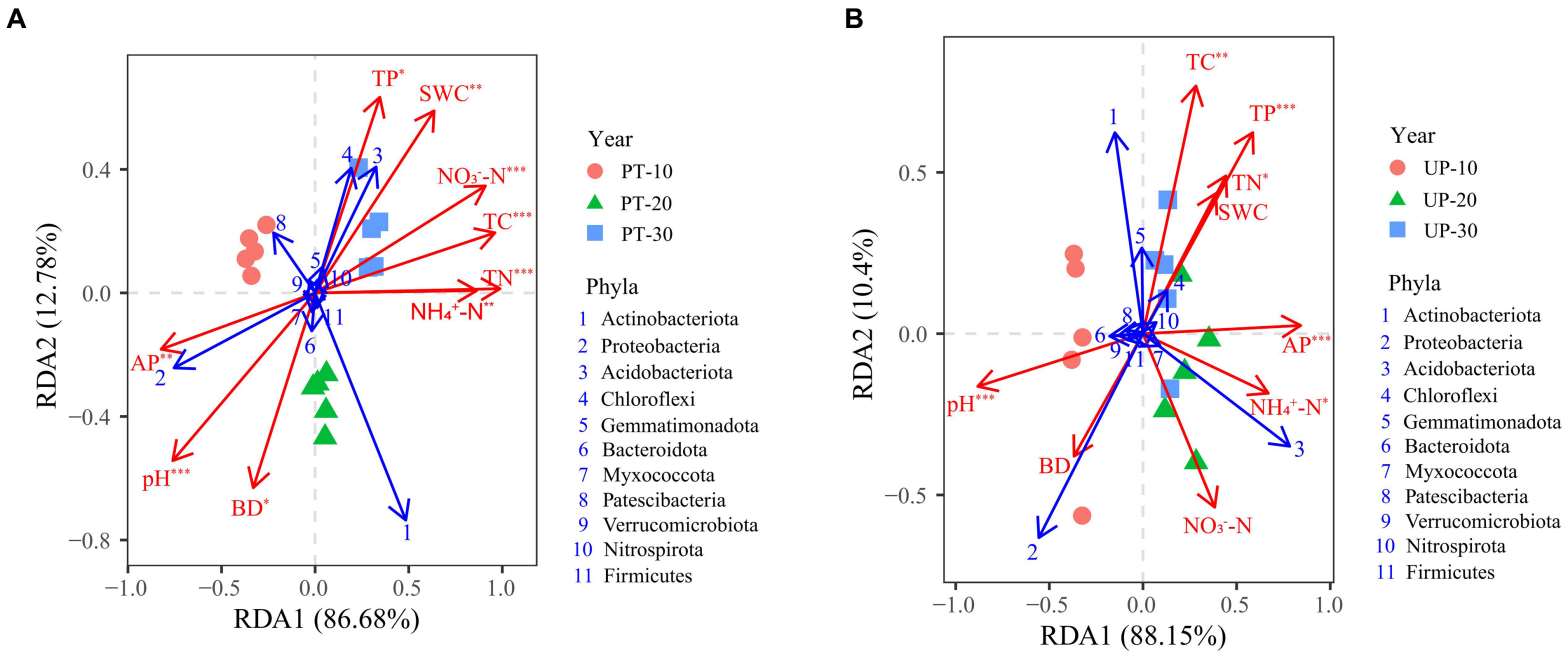
Redundancy analyses (RDA) between soil environmental factors (red arrows) and bacterial community structure at the phylum level (relative abundance > 0.1%, blue arrows) of PT **(A)** and UP **(B)**. Significant factors were identified by Monte Carlo permutation tests and are labeled as ^*^p < 0.05, ^**^p < 0.01, and ^***^p < 0.001.

The TC, TN, NH_4_^+^-N, NO_3_^−^-N, TP, AP, BD, pH, and SWC were the main factors influencing the bacterial community structure for PT, according to the results of the redundancy analysis and Monte Carlo permutation tests (Figure 5A; Supplementary Table S3). The TC, TN, TP, AP, NH_4_^+^-N, and pH all significantly affected the bacterial community structure of UP (Figure 5B; Supplementary Table S3). In addition, the soil pH, TC, TN, NH_4_^+^-N, and AP significantly (*p* < 0.05) correlated with the diversity index of the bacterial communities of PT and UP (Supplementary Table S4).

### Differentiating bacterial beta diversity determinants

**Figure 6:**
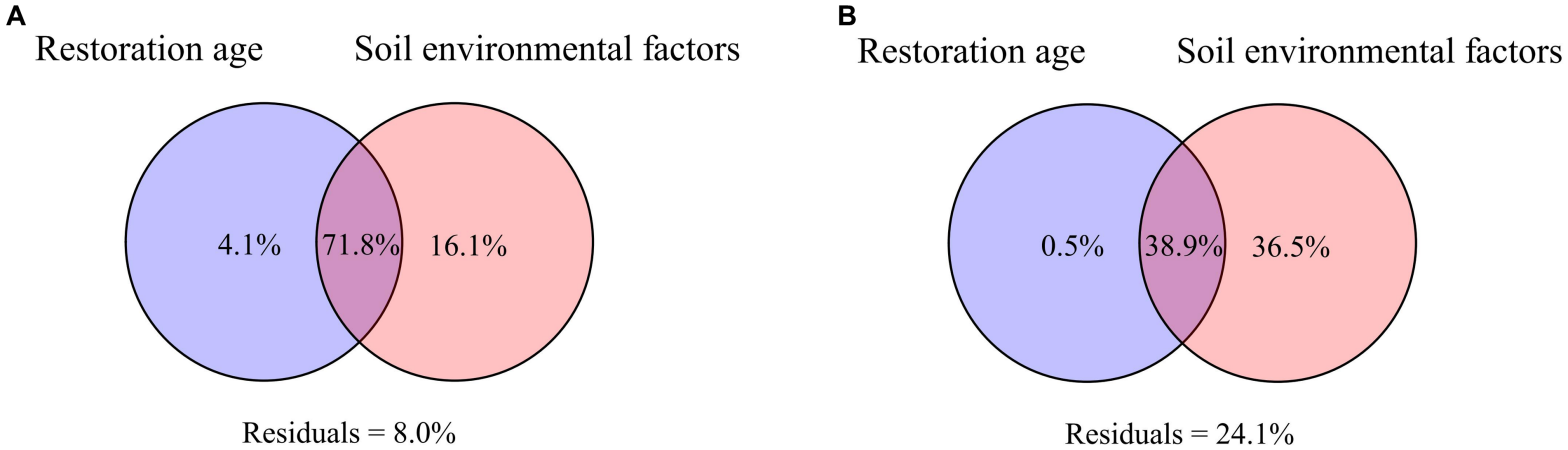
Variation-partitioning analysis (VPA) illustrates the forest stand age and soil environmental factors on bacterial community for PT **(A)** and UP **(B)**.

VPA was applied to evaluate the relative contributions of soil and vegetation factors to changes in the soil microbial communities. In both PT and UP, the changes in the bacterial communities were primarily driven by soil factors, according to the results of VPA, which explained 16.1% of the variation for PT (Figure 6A) and 36.5% of the variation for UP (Figure 6B). The variation in bacterial communities could only be partially explained by variations in restoration ages. For instance, only 4.1% of the variation in the bacterial community for PT could be explained by the difference in restoration age, which explained only 0.5% of the variation for UP (Figure 6). Soil factors and vegetation ages jointly explained 71.8% and 38.9% of the variation for PT and UP, respectively (Figure 6).

**Figure 7:**
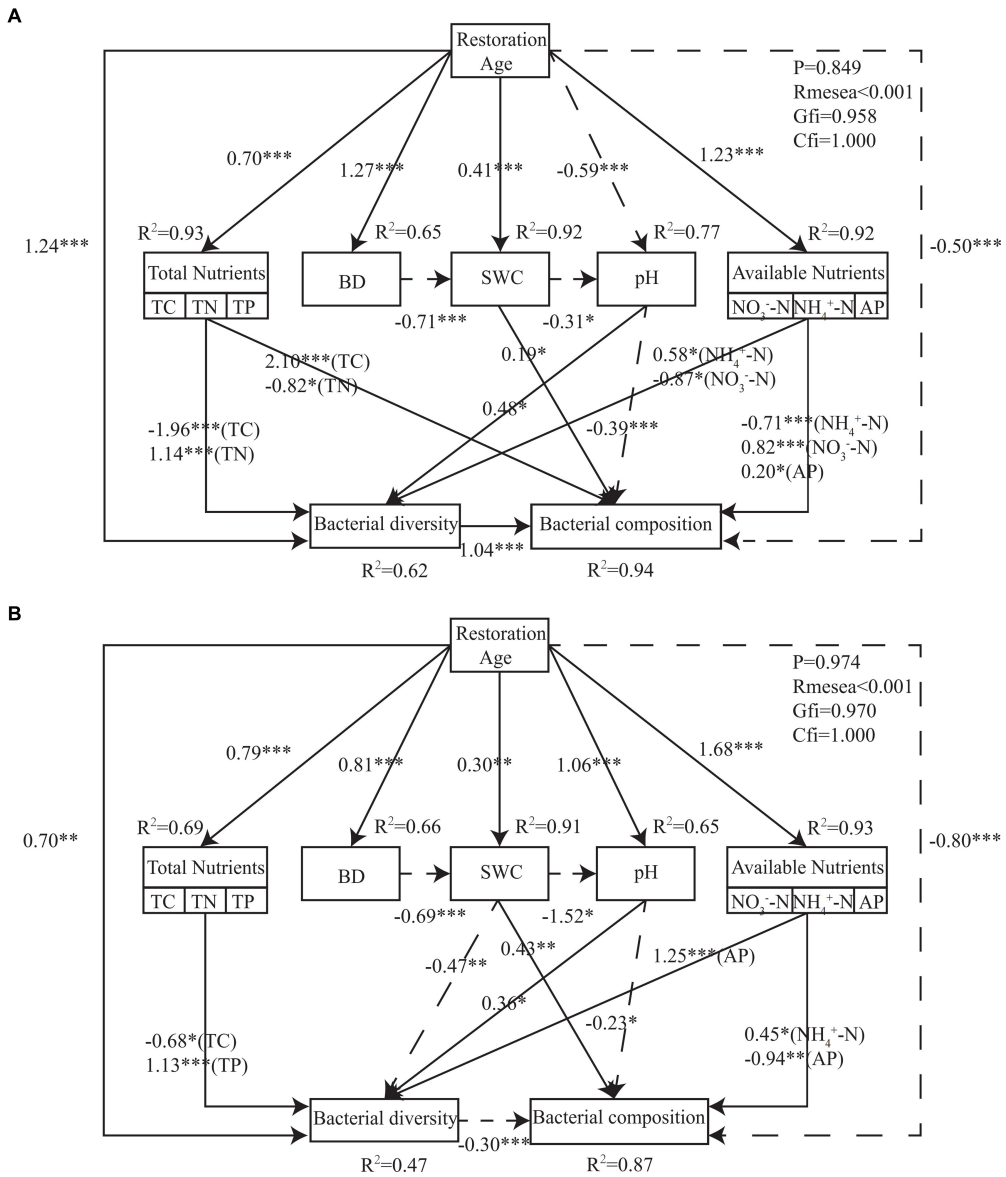
Structural equation model describing the effects of restoration age, soil physical and chemical properties on bacterial community diversity and structure for PT **(A)** and UP **(B)**. Solid lines indicate positive effects, and dashed lines indicate negative effects. Numbers on lines are standardized direct path coefficients. The significant paths are reported as follows: *p < 0.05; **p  < 0.01; ***p  < 0.001.

Interactions between soil environmental factors, bacterial community diversity, and bacterial community structure were assessed using structural equation model (SEM). The findings demonstrated that the final SEM successfully fit PT and UP, *p* ≥ 0.849, root mean square error of approximation (RMSEA) < 0.001, goodness-of-fit index (GFI) ≥ 0.958, and comparative fit index (CFI) = 1.000 (Figure 7). For both PT and UP, the restoration age directly positively impacted the bacterial community diversity and directly negatively impacted the bacterial community structure. Through its effects on soil total nutrient (TC, TN, and TP) and available nutrient (NH_4_^+^-N, NO_3_^−^-N, and AP) variables, it also indirectly had a positive impact on the diversity and structure of the bacterial communities. The structure of bacterial communities was negatively impacted by soil pH and positively impacted by SWC for both PT and UP (Figure 7). Additionally, the integrated fertility index (IFI) at 30 years of restoration was 2.683 and 2.447 for PT and UP, respectively, which are much higher than those at 10 and 20 years (Table 3).

**Table 3:** Integrated fertility index (IFI) for PT and UP at different restoration ages.

Forest type	Restoration age	F1	F2	IFI
PT	10 years	−2.164	−0.275	−1.767
	20 years	−0.845	1.669	−0.317
	30 years	3.537	−0.530	2.683
UP	10 years	−2.961	−0.177	−2.295
	20 years	0.474	0.483	0.476
	30 years	3.265	−0.156	2.447

## Discussion

### Effects of forest restoration type and age on soil physicochemical properties

Different vegetation types and restoration ages can change the physical structure and chemical properties of the soil (Fu et al., 2010; Zhao et al., 2013). In this study, significant differences in soil bulk density were found between the two forest types (*p* < 0.05) and restoration ages (*p* < 0.001), i.e., the soil bulk density was significantly lower for both the coniferous forest (*Pinus tabuliformis,* PT) and broad-leaved forest (*Ulmus pumila*, UP) after 30 years of restoration than after 10 and 20 years of restoration. These findings are consistent with those of earlier studies, in which the soil bulk density decreased as the restoration age increased (Fu et al., 2010; Yang and Wang, 2013; Liu et al., 2019). As vegetation restoration progresses, more roots penetrate the soil, increasing porosity and lowering soil bulk density (Feng et al., 2019). Moreover, at 30 years, both PT and UP had the highest TC, TN, NH_4_^+^-N, NO_3_^−^-N, and TP contents, but had lower pH values compared with at 20 and 10 years. These findings, which are consistent with those of earlier studies, demonstrate that restoration activities require long-term periods (>20 years) to considerably increase soil TN and SOC (Shrestha and Lal, 2010). This is primarily because the buildup of litter, leaves, roots, and other above-ground biomass is required to aid in the production of humus (Frouz et al., 2007; Mukhopadhyay et al., 2014). Other researchers have also reported a significant change in soil pH due to restoration activities (Zipper et al., 2013).

### Bacterial community impacted by forest type and restoration age

*Proteobacteria*, *Actinobacteria*, *Acidobacteria*, and *Chloroflexi* were the most prevalent bacterial phyla in our study across the two forest types and three restoration ages. This result is generally consistent with that of an earlier study on the soils of mining areas in northern China (Chen et al., 2020). In forest soils, earlier studies have also found that *Proteobacteria* are the most abundant phylum (Hui et al., 2014; Miyashita, 2015; Deng et al., 2018). In addition, *Proteobacteria* were more abundant than *Actinobacteria*, consistent with the findings of other studies (Qiu et al., 2012; Guo et al., 2019). However, while the bacterial community compositions were similar, the structure significantly changed in both PT and UP in our study; e.g., the *Proteobacteria* abundances decreased for both PT and UP as the restoration ages increased, but the abundance of *Acidobacteria* showed the opposite trend and was negatively correlated with soil pH (Chu et al., 2010; Griffiths et al., 2011; Guo et al., 2019).

The bacterial community alpha diversity was changed to some extent by the forest restoration activities; both PT and UP had higher alpha diversity after 30 and 20 years of restoration than at 10 years. This could primarily be attributed to the substantial amount of litter generated by the prolonged establishment of *Pinus tabuliformis* and *Ulmus pumila*, which encouraged microbial decomposition (Guo et al., 2019). The soil TN and SOC contents increased (Shrestha and Lal, 2010) with increasing restoration age, which provided sufficient energy and increased microbial activity levels (Jia et al., 2005). The two-way ANOVA results indicated that the bacterial community structure and diversity of UP differed from those of PT, which might be due to the forest types affect leaf litter quality and quantity, which can drastically change the chemical characteristics of the soil and have an impact on the microbiological activities in the soil (Nawaz et al., 2013; Šnajdr et al., 2013; Deng et al., 2018). These results supported our proposed hypothesis.

The complex relationships between microorganisms can be analyzed using co-occurrence network analysis, proving the mathematical validity of microbial community aggregation (Toju et al., 2016; Zhang et al., 2022). For both PT and UP, the microbial networks significantly varied among the different restoration ages in the microcosms, and variations in the PT and UP were noted (Figure 3). A larger number of links indicates a more complex network, and more complex networks are typically more stable (Liu et al., 2022b). According to this study, the number of links in both the PT and UP microbe networks increased with increasing restoration age, which implied that a higher restoration age may be beneficial to the soil microbiota’s complexity. The increase in the percentage of positive links with increasing restoration age was also found in this study. These findings suggest that cooperation may be a key mode of interaction between bacteria in relatively high-quality soil.

### Factors driving soil bacterial community

In severely disturbed soils, the physical, chemical, and biological components of the soil are interconnected and play crucial roles in the creation of a functioning soil system (Rowland et al., 2009; Feng et al., 2019). The physicochemical characteristics of the soil are key factors in soil microbial development (Orgiazzi et al., 2012). Microorganisms can also reveal soil quality and are crucial to the functioning of the environment (Damon et al., 2012; Guo et al., 2019). Microorganisms’ diversity and composition typically depend on environmental factors, such as soil nitrogen availability (Jesus et al., 2009). In this study, most of the considered soil environmental factors affected the bacterial community. The diversity and organization of the bacterial communities in both PT and UP were mostly influenced by TC, TN, NH_4_^+^-N, TP, AP, and pH. Researchers have also reported that the effects of soil environmental factors on microbial community result from their synergism, rather than being governed by a single component alone (Deng et al., 2018; Guo et al., 2019).

Structural equation model (SEM) was also used to evaluate the interactions among the soil physicochemical factors, bacterial composition and diversity, which demonstrated that, with increasing restoration age, the soil quality improved, as evidenced by the positive effects on nutrients, BD, and SWC and decrease in soil pH. This finding is in line with the findings of earlier studies showing that artificial restoration of vegetation may considerably enhance the soil’s nutritional condition and soil quality in mining areas (Zhao et al., 2023) and that the improvement effect becomes more pronounced over time (Shrestha and Lal, 2010; Wang et al., 2013; Zipper et al., 2013). Moreover, our findings demonstrate that the duration of the restoration activities positively affected the diversity, but negatively affected the composition of bacterial communities for both PT and UP. This might have been due to the different vegetation types and restoration durations resulting in different rates of litter decomposition and nutrient release, which impact microorganism diversity (Vesterdal et al., 2012; Nawaz et al., 2013; Zhao et al., 2013). Jia et al. (2005) reported that the plentiful soil TN which providing enough energy can increase microbial activity levels. Moreover, with the increase in the restoration age, soil carbon contents increase, which also can affect on soil bacteria (Jia et al., 2005; McGeea et al., 2019). In addition, soil pH can be changed after the restoration of vegetation. It was found to be decreased as the restoration ages increasing in this study, which is consistent with previous report in mining area (Zipper et al., 2013). And soil pH then effect on soil bacteria. Studies reported that the abundance of *Acidobacteria* was negatively correlated with soil pH (Griffiths et al., 2011; Guo et al., 2019). Researchers also found soil pH soil pH may have an impact on amino acid metabolism, which has a strong negative correlation with proteobacteria (Zhao et al., 2023). Studies suggested that differences in soil bacterial communities in various locations were largely caused by differences in soil pH among various vegetation types (Shi et al., 2021).

## Conclusion

In this study, bacterial community structure and diversity were sensitive to the forest restoration type and age. Significant differences among the different forest types and restoration ages were found in terms of soil physical, chemical, and microbial properties in the open-pit coal mine dumps. In general, at 30 years, both coniferous forest (PT) and broadleaf forest (UP) had higher TC, TN, NH_4_^+^-N, NO_3_^−^-N, and TP contents, but had lower pH values than at 10 and 20 years. The soil quality notably improved with increasing restoration age for both PT and UP, as evidenced by the positive effects on nutrients, BD, and SWC and decrease in soil pH. In addition, the soil TC, TN, NH_4_^+^-N, TP, AP, and pH were the primary soil environmental factors driving the bacterial community structure of PT and UP. The integrated fertility index (IFI) at 30 years of restoration was much higher for PT and UP than at 10 and 20 years. Overall, this study advances knowledge of how bacterial communities react to various types and ages of forest restoration in open-pit coal mine dumps in semiarid areas.

## Data availability statement

The data presented in this study are deposited in the National Center for Biotechnology Information (NCBI) Sequence Read Archive database (SRA) received the sequencing data, accession number PRJNA976285.

## Author contributions

At all stages, SL, YG, JC, JL, and HZ provided feedback on the manuscript. SL, JL, and HZ conceived and designed the study. YG contributed materials and analysis tools. SL and JC contributed to paper preparation. All authors contributed to the article and approved the submitted version.

## Funding

This study was supported by the National Natural Science Foundation of China (grant numbers U1910207, U22A20557, and 41401618).

## Conflict of interest

The authors declare that the research was conducted in the absence of any commercial or financial relationships that could be construed as a potential conflict of interest.

## Publisher’s note

All claims expressed in this article are solely those of the authors and do not necessarily represent those of their affiliated organizations, or those of the publisher, the editors and the reviewers. Any product that may be evaluated in this article, or claim that may be made by its manufacturer, is not guaranteed or endorsed by the publisher.

## Footnotes

^1^https://www.bioincloud.tech/

^2^https://gephi.org/
