# Supplementary material for: Responses of soil bacterial community structure to different artificially restored forests in open-pit coal mine dumps on the loess plateau, China
Source: Front Microbiol. 2023 Jul 28;14:1198313. doi: 10.3389/fmicb.2023.1198313 (PMC10416249; doi:10.3389/fmicb.2023.1198313)
Supplement: Supplementary file 1 [file Data_Sheet_1.pdf]

Table S1 Relative abundance of dominant phyla in different forest types and ages.

Forest type	Stand age	*Actinobacteriota*	*Proteobacteria*	*Acidobacteriota*	*Chloroflexi*
PT	10 years	21.63±0.646b	38.85±1.024a	12.04±0.528c	11.17±0.561b
	20 years	32.29±0.499a	31.86±0.554b	14.02±0.197b	10.42±0.077b
	30 years	32.56±1.335a	21.85±0.554c	19.59±0.611a	16.27±0.114a
UP	10 years	30.82±1.355a	27.65±0.843a	15.83±0.794b	12.41±0.350b
	20 years	30.45±0.928a	23.54±0.279b	22.10±0.679a	13.28±0.0443a
	30 years	30.22±0.514a	23.43±0.283b	20.94±0.617a	13.78±0.179a
Summary of ANOVA (*P* values)					
Forest type		0.063	<0.001	<0.001	0.003
Restoration age		<0.001	<0.001	<0.001	<0.001
Forest type x restoration age		<0.001	<0.001	<0.001	<0.001

Note: Values are mean±SE, n=5. Statistical significance was assessed by one-way ANOVA followed by LSD multiple comparison test. Different lowercase letters in the same column indicate significant difference ($p < 0.05$).

Table S2 Dissimilarity test of bacterial community structure for different forest types and ages.

Forest type and restoration age	PERMANOVA		ANOSIM		MRPP	
	R^2	*p* value	R	*p* value	R	*p* value
PT all ages	0.859	**0.001**	1.00	**0.001**	0.731	**0.001**
PT-10 vs PT-20	0.894	**0.016**	1.00	**0.010**	0.626	**0.008**
PT-10 vs PT-30	0.962	**0.008**	1.00	**0.009**	0.758	**0.005**
PT-20 vs PT-30	0.882	**0.010**	1.00	**0.009**	0.604	**0.011**
UP all ages	0.471	**0.004**	0.78	**0.001**	0.508	**0.001**
UP-10 vs UP-20	0.813	**0.009**	1.00	**0.007**	0.526	**0.009**
UP-10 vs UP-30	0.807	**0.004**	1.00	**0.012**	0.524	**0.009**
UP-20 vs UP-30	0.335	**0.039**	0.36	**0.039**	0.145	**0.046**

Table S3 Monte Carlo permutation tests the relationships of bacterial community structure with soil factors.

Soil factors	PT		UP	
	R^2	P value	R^2	P value
SWC	0.3165	0.105	0.7392	0.002**
PH	0.7736	0.001***	0.8623	0.001***
TC	0.5819	0.006**	0.9432	0.001***
TN	0.4056	0.041*	0.9491	0.001***
TP	0.684	0.001***	0.503	0.014*
NO_3^--N	0.3516	0.066	0.9354	0.001***
NH_4^+N	0.4366	0.037*	0.7243	0.002**
AP	0.6728	0.001***	0.6976	0.002**
BD	0.2618	0.178	0.489	0.016*

Note: *** $p < 0.01$; ** $p < 0.01$; * $p < 0.05$.

Table S4 The spearman correlations between diversity index of bacterial community structure with soil factors.

	**SWC**	**pH**	**TC**	**TN**	**TP**	**NO_3^--N**	**NH_4^+-N**	**AP**	**BD**
PT									
Shannon	0.136	-0.438	0.786**	0.636*	0.132	0.764**	0.764**	-0.850**	-0.071
ACE	0.218	-0.422	0.793**	0.679**	0.146	0.807**	0.768**	-0.929**	-0.175
Chao1	0.057	-0.282	0.704**	0.618*	-0.071	0.754**	0.693**	-0.871**	-0.007
UP									
Shannon	0.729**	-0.734**	0.482	0.482	0.754**	0.139	0.704**	0.718**	-0.632*
ACE	0.804**	-0.829**	0.575*	0.671**	0.854**	0.096	0.721**	0.807**	-0.779**
Chao1	0.768**	-0.788**	0.607*	0.686**	0.832**	0.021	0.650**	0.743**	-0.743**

Note: *** $p < 0.01$; ** $p < 0.01$; * $p < 0.05$.
